# Lipoprotein(a) as a predictor of poor collateral circulation in patients with chronic stable coronary heart disease

**DOI:** 10.1590/1414-431X20175979

**Published:** 2017-07-10

**Authors:** Y. Fan, J.-S. Hu, F. Guo, Z.-B. Lu, H. Xia

**Affiliations:** 1 Department of Cardiology, Renmin Hospital, Wuhan University Hubei General Hospital, Wuhan, Hubei Province, China; 2 Department of Cardiology, The Fifth Hospital of Wuhan and Affiliated Guangci Hospital, Wuhan University, Wuhan, Hubei Province, China

**Keywords:** Lipoprotein(a), Coronary collateral circulation, Predictor

## Abstract

As a mechanism compensating for obstructive coronary artery disease, coronary collateral circulation (CCC) has attracted cardiologists for a long time to explore its potential impact. In the present study, Chinese patients suffering from ≥95% coronary stenosis, as diagnosed by angiography, have been investigated for the correlation between CCC and lipoprotein(a) [Lp(a)] levels. A cohort of 654 patients was divided into four categories according to Rentrop grades 0, 1, 2, and 3. Lp(a) levels were divided into model 1, discretized with critical values of 33 and 66%, and model 2, discretized with a cutoff value of 30.0 mg/dL. Furthermore, we evaluated the correlation between CCC and serum Lp(a) levels. The four groups had significantly different Lp(a) levels (25.80±24.72, 18.99±17.83, 15.39±15.80, and 8.40±7.75 mg/dL; P<0.001). In model 1, concerning R0, the risk in the third Lp (a) tertile (OR=3.34, 95%CI=2.32-4.83) was greater than that in the first tertile. In model 2, concerning R0, the risk in Lp(a) >30.0 group (OR=6.77, 95%CI=4.44-10.4) was greater than that of Lp(a) <30.0 mg/dL. The worst condition of CCC can be predicted independently by Lp(a) levels. In addition to clinical usage, Lp(a) levels can also be utilized as biological markers.

## Introduction

Lipoprotein(a) [Lp(a)] is a combination of low-density lipoprotein (LDL) and a glycoprotein [apolipoprotein(a)], a homolog of plasminogen, linked to each other by a sulfhydryl bridge (1). A high concentration of serum Lp(a) has been identified as a risk factor for atherosclerosis, restenosis after angioplasty, ischemic heart disease, and stroke (2–5). Although not yet clearly understood, the putative mechanism of atherothrombosis mediated by Lp(a) is multifactorial including endothelial dysfunction (6–8).

Coronary artery disease (CAD) is one of the most common causes of morbidity and mortality in developed countries. Coronary collateral growth is a major process in patients with CAD. Moreover, the development of collateral vessels is a physiological adaption to severe coronary artery narrowing and/or occlusion for myocardium to circumvent ischemia (9,10). Thus, the coronary collateral circulation (CCC) can protect and preserve the myocardium from episodes of ischemia, enhance residual myocardial contractility, and reduce angina symptoms and cardiovascular events (11–15). Previous studies have suggested that the severity of coronary artery stenosis and myocardial hypoxia are the potential causes of CCC development (16,17). However, the underlying physiological and pathological factors influencing the development of CCC remain unclear. Thus, to improve the survival following coronary narrowing/occlusion, we must gain further insight into these factors.

Previous studies show that intact vascular endothelium and endothelial function are essential for coronary collateral growth (18). Therefore, factors that contribute to vascular dysfunction should theoretically lead to poor CCC. However, the correlation between Lp(a) and collateral circulation is yet an enigma. Aras et al. (19) observed a strong negative correlation between Lp(a) and vascular endothelial cell growth in only 60 CAD patients. Therefore, the current study was undertaken to assess whether the Lp(a) concentration was associated with the extent of angiographically visible coronary collateral vessels in patients with high-grade coronary artery stenosis or occlusion in a large Chinese cohort treated for the disease.

## Material and Methods

### Study population

**Figure 1.**
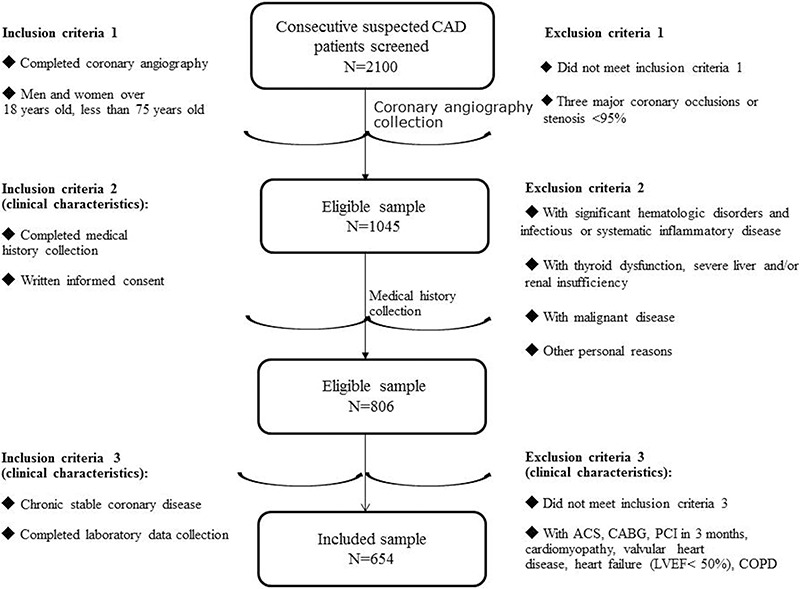
Flow diagram of patient recruitment. CAD: coronary artery disease; ACS: acute coronary syndrome; CABG: coronary artery bypass grafting; PCI: percutaneous coronary intervention; LVEF: left ventricular ejection fraction; COPD: chronic obstructive pulmonary disease.

All patients who underwent coronary angiography at our institution (Renmin Hospital of Wuhan University, Hubei, China) from March 2012 to July 2015 and were found to have at least one major coronary occlusion or a stenosis of ≥95% with thrombolysis in myocardial infarction (TIMI) grade 1 anterograde-flow were screened for eligibility. Exclusion criteria included: 1) any known inflammatory or infectious disease, confirmed or suspected cancer, 2) treatment with steroids, immune suppressive drugs or non-steroidal anti-inflammatory drugs except low-dose aspirin, 3) acute coronary syndrome within the previous 6 months, 4) percutaneous coronary intervention within the previous 3 months, 5) history of coronary artery bypass operation, 6) chronic heart failure (EF<50%), cardiomyopathy, valvular heart disease, 7) pulmonary heart disease, 8) age >75 years, 9) severe liver and kidney dysfunction. The study protocol was approved by the Renmin Hospital of Wuhan University Ethics Committee, and written consent was obtained from all the subjects (Figure 1).

### Coronary angiography and collateral scoring

Routinely, Judkin's method that did not use nitroglycerin was applied to perform coronary angiography. The biplane-mode computerized quantitative angiography was employed to measure the percentage stenosis diameter (Philips DCI, The Netherlands). Two cardiologists, who were blind to the patients' features, reviewed the angiograms and ranked the coronary collaterals in accordance with the Rentrop classification (20) (Rentrop scoring system: 3 = complete collateral filling of the epicardial artery; 2 = partial collateral filling of the epicardial artery; 1 = filling of the small side branches; 0 = no visible filling of any collateral channels. Fifty coronary angiograms were selected by random sampling to determine the intra- and inter-observer agreements of coronary collateral grades; joint reading was applied to resolve disagreements.

### Risk factor assessment

Patient data, including physical activity status, alcohol consumption, and smoking, were collected through direct interview. In the current study, daily alcohol consumption referred to the patient consuming minimally one drink per day, and smoking referred to at least one cigarette daily. Furthermore, their medical records were ascertained for previous MI and PCI reports. The experiment followed the guidelines from the American Diabetes Association to define diabetes (21). Hypertension was defined as using anti-hypertensive medication in the previous 2 weeks or displaying a diastolic blood pressure of 90 mmHg and a systolic blood pressure of 140 mmHg, and the weight (kg) divided by height^2^ was used to calculate the body mass index (BMI, kg/m^2^).

### Laboratory measurement

Before coronary angiography, all participants were subjected to a 12-h overnight fast, followed by collection of venous blood samples. Proteolysis was minimized with ethylene-diamine-tetra-acetic acid (EDTA). Under such conditions plus sodium azide, venous sera from a peripheral vessel were collected and stored at –70°C, for further analysis. Triglyceride (TG), Lp(a), total cholesterol (T-chol), and high-density lipoprotein (HDL) cholesterol (HDL-C) were measured. Friedewald equation was used to calculate the serum levels of low-density lipoprotein (LDL) cholesterol. To refrain from possible variations caused by long-term storage, we gathered the blood sample and immediately estimated the Lp(a) parameters. One-step sandwich enzyme-linked immunosorbent assay (ELISA) commercial test kits (Immuno GMBH, Germany) were used to confirm Lp(a) concentration. All samples were analyzed twice to obtain their mean value.

### Statistical analyses

SPSS 19.0 for Windows (SPSS Inc., USA) was employed for the statistical analyses. The continuous variables are reported as means±SD, and the categorical variables are reported as percentages. The non-normally distributed continuous variables were compared by Mann-Whitney U test while normally assigned variables were compared by Student's *t*-test for two groups and ANOVA for three groups or more. Chi-square test was employed to compare categorical data among groups. Univariate analyses were carried out to identify the possible predictors of CCC, with a threshold of P<0.05. Predictors were then used as independent variables in regression models. The correlation between the coronary collateral scoring and independent variables was tested by conducting multivariate logistic regression analysis.

## Results

### Baseline characteristics

Baseline data are summarized in Table 1. A cohort of 654 patients who fulfilled the eligibility criteria was included in the study. The four groups showed no significant difference concerning gender. When the Rentrop grade increased gradually, the Lp(a) values declined (P<0.001). Compared with Rentrop 0, and 1, the frequency of diabetes was lower in Rentrop 2 and 3 (P=0.001). At different collateral levels, the cardiovascular medications had similar applications, and all other cardiovascular risk factors had similar prevalence.

**Table 1.** Baseline characteristics of the patients.

Variables	Rentrop collateral classification				P
	0 (n=44)	1 (n=91)	2 (n=232)	3 (n=287)	
Clinical characteristics					
Age (years)	58.82±9.57	56.21±9.66	57.01±9.83	55.32±9.40	0.064
Male (n, %)	32 (72.73%)	69 (75.82%)	188 (81.03%)	236 (82.23%)	0.321
BMI (kg/m2)	26.54±2.96	26.05±3.12	25.78±3.32	26.13±3.03	0.393
Current smokers (n, %)	13 (29.55%)	30 (32.97%)	84 (36.21%)	102 (35.54%)	0.819
Drinking (n, %)	7 (15.91%)	15 (16.48%)	58 (25.00%)	58 (20.21%)	0.247
Hypertension (n, %)	32 (72.73%)	59 (64.84%)	152 (65.52%)	182 (63.41%)	0.682
Diabetes mellitus (n, %)	20 (45.45%)	38 (41.76%)	68 (29.31%)	63 (21.95%)	0.000
Previous MI (n, %)	13 (29.55%)	21 (23.08%)	67 (28.88%)	77 (26.83%)	0.740
Previous PCI (n, %)	13 (29.55%)	26 (28.57%)	47 (20.26%)	59 (20.56%)	0.215
Laboratory findings					
LP(a) (mg/dL)	25.80±24.72	18.99±17.83	15.39±15.80	8.40±7.75	0.000
LP(a) (Lg)	5.13±0.95	4.81±0.98	4.42±1.22	3.93±1.12	0.000
Total cholesterol (mg/dL)	4.20±1.23	4.08±1.01	4.17±1.21	4.16±1.23	0.926
HDL	1.05±0.32	1.00±0.24	1.02±0.34	1.03±0.34	0.881
LDL	2.52±1.05	2.49±0.88	2.50±0.95	2.54±1.04	0.952
Triglycerides (mg/dL)	1.95±1.20	1.80±0.94	1.91±1.10	1.97±1.30	0.668
C-reactive protein (mg/L)	81.23±15.66	78.41±16.32	77.02±14.62	75.48±15.84	0.082
Fasting glucose (mg/dL)	6.05±2.07	5.73±1.42	5.87±1.78	5.83±1.71	0.778
Cardiovascular medication					
Aspirin	42 (95.45%)	89 (97.80%)	224 (96.55%)	280 (97.56%)	0.482
Beta-blockers	40 (90.91%)	82 (90.11%)	202 (87.07%)	237 (82.58%)	0.203
ACE-Is or ARBs	21 (47.73%)	26 (28.57%)	82 (35.34%)	103 (35.89%)	0.185
Calcium channel blockers	15 (34.09%)	35 (38.46%)	76 (32.76%)	103 (35.89%)	0.797
Nitrates	41 (93.18%)	86 (94.51%)	209 (90.09%)	264 (91.99%)	0.765
Statin	31 (70.45%)	54 (59.34%)	129 (55.60%)	157 (54.70%)	0.238

Data are reported as means±SD or the number (%) of patients. BMI: body mass index; MI: myocardial infarction; PCI: percutaneous coronary intervention; LP(a): lipoprotein(a); Lg: logarithm; HDL: high-density lipoprotein; LDL: low-density-lipoprotein; ACE-Is: angiotensin converting enzyme inhibitors; ARBs: angiotensin receptor blockers. Statistical analysis was done with ANOVA or the chi-square test.

### Coronary angiographic and logistic regression analysis

The coronary angiographic results of patients are summarized in Table 2. The diseased vessels did not display a significant difference regarding number among the four groups. The frequency of the right coronary artery (RCA) increased with increasing Rentrop grade (P<0.001).

**Table 2.** Coronary angiographic findings of the patients.

Variables	Rentrop classification				P
	0 (n=44)	1 (n=91)	2 (n=232)	3 (n=287)	
LAD	19 (43.18%)	43 (47.25%)	88 (37.93%)	93 (32.40%)	0.058
LCX	17 (38.64%)	42 (46.15%)	88 (100%)	90 (96.05%)	0.065
RCA	11 (25%)	34 (37.36%)	118 (50.86%)	195 (67.94%)	<0.001
One-vessel disease	41	68	179	210	
Two-vessel disease	3	20	45	69	
Three-vessel disease	0	3	8	8	0.149

Data are reported as number (%). LAD: left anterior descending artery; LCX: left circumflex artery; RCA: right coronary artery. Statistical analysis was done with the chi-square test.

The models below had been applied to categorize Lp(a) levels: in model 1, Lp(a) group was discretized with critical values of 33 and 66%; in model 2, it was discretized with the cutoff value of 30.0 mg/dL. The association between Rentrop grades and different Lp(a) levels was assessed by multiple-factor and single-factor logistic regression. The multiple-factor logistic regression was applied to correct RCA, BMI, DM, gender, age, smoking, and NDV (number of diseased vessels). As displayed in model 1 of single-factor logistic regression, for the poor collateral circulation group, the risk in the third Lp(a) tertile (OR=3.38, 95%, CI=2.36–4.85) was greater than that in the first Lp(a) tertile. Nevertheless, its difference was not significant in the second tertile. As shown in Table 3, the single-factor analysis outcomes were similar to those of the multiple-factor analysis, and model 1 displayed results similar to model 2.

**Table 3.** Multivariate logistic regression for the presence of coronary collaterals according to lipoprotein(a) levels.

	n	Rentrop				Univariate		Multivariate	
		0	1	2	3	Odds ratio (95%CI)	P	Odds ratio (95%CI)	P
Lp(a) - Model 1									
<7.17	218	13 (5.96)	20 (9.17)	70 (32.11)	115 (52.75)	1		1	
7.17-13.50	218	6 (2.75)	19 (8.72)	72 (33.03)	121 (55.50)	0.85 (0.59-1.22)	0.377	0.84 (0.58-1.21)	0.349
>13.50	218	25 (11.47)	52 (23.85)	90 (41.28)	51 (23.39)	3.38 (2.36-4.85)	<0.001	3.34 (2.32-4.83)	<0.001
Lp(a) - Model 2									
≤30.00	571	30 (5.25)	63 (11.03)	191 (33.45)	287 (50.26)	1		1	
>30.00	83	14 (16.87)	28 (33.73)	41 (49.40)	0 (0.00)	6.87 (4.41-10.7)	<0.001	6.77 (4.44-10.4)	<0.001

Data are reported numbers (%). The final models were adjusted for age, gender, body mass index, current smoking, diabetes mellitus, and number of diseased vessels. Lp(a): lipoprotein(a); CI: confidence interval.

## Discussion

In the present Chinese cohort of 654 patients with coronary artery occlusion or a stenosis of ≥95% TIMI grade 1 anterograde-flow on their angiograms, we assessed the association between the Lp(a) concentrations and angiographically visible coronary collateral formation. Our findings indicated that 1) elevated Lp(a) levels were associated with a significant impairment in coronary collateralization, and 2) the baseline serum Lp(a) level was found to be an independent predictor of poor collateral development in CAD patients.

Previous studies have proposed several factors to be related to coronary collateral development, such as genetic factors, age, degree of coronary artery stenosis, the presence of total occlusion, myocardial ischemia, physical exercise, smoking, body mass index, hyperlipidemia, hyperhomocysteinemia, diabetes, and inflammation (17,20–23). In the present study, we found that diabetes and the number of diseased vessels were associated with CCC. However, the underlying physiological and pathological factors influencing the development of CCC have not yet been completely described.

Recent studies have demonstrated that the collateral response of a patient is a complex mixture of two closely linked processes, angiogenesis and arteriogenesis. Angiogenesis involves the coordinated migration, proliferation, and differentiation of endothelial cells and pericytes from existing vascular beds. On the other hand, arteriogenesis is the growth of muscular arteries requiring similar events regulated by endothelial cells and smooth muscle cells from pre-existing arteries (24). Angiogenesis or arteriogenesis, an intact vascular endothelium, and endothelial function are vital for the process of collateral growth adaptation, as well as vascular endothelial dysfunctions are speculated to be one of the major factors disturbing this process (25).

Hitherto, few studies found that the high serum Lp(a) concentration was associated with endothelial dysfunctions. Morishita et al. (26) demonstrated that high serum Lp(a) concentration affected the collateral formation in the Lp(a) transgenic mouse hind limb ischemia model. In their study, in addition to the inhibition of activation of transforming growth factor (TGF-β), Lp(a) inhibited angiogenesis, as it stimulated the proliferation of vascular smooth muscle cells. Aras et al. (19) established a strong negative correlation between Lp(a) and vascular endothelial cell growth factor (VEGF). High level of Lp(a) negatively affects the formation of coronary collateral vessels in humans by reducing the production or bioactivity of VEGF; however, this was found only in 60 patients. In the present Chinese cohort of 654 patients, we found an inverse graded association between Lp(a) and the presence of coronary collaterals. When Lp(a) was grouped into two types of models, the risks of R0 were higher in the third Lp(a) tertile than in the first Lp(a) tertile in both the single- and multiple-factor logistic regression analyses. Taken together, these data suggest that the high degree of inflammation might be associated with impaired CCC vessels.

However, there were several limitations in our study. First, CCC formation was assessed by coronary angiography only, while intravascular strategies might be more precise. Second, because this was an observational study, potential mechanisms were not elucidated. Third, the diameter of the CCC connection was not analyzed to evaluate its function and the outcomes of the patients.

In conclusion, the present data demonstrated that serum Lp(a) level was a valuable and independent predictor of poor CCC as assessed using the Rentrop grading system.
